# Neural correlates of word processing influenced by painful primes

**DOI:** 10.1371/journal.pone.0295148

**Published:** 2024-01-19

**Authors:** Christoph Brodhun, Eleonora Borelli, Thomas Weiss

**Affiliations:** 1 Department of Psychology, Clinical Psychology, Friedrich Schiller University, Jena, Germany; 2 Department of Medical and Surgical Sciences, University of Modena and Reggio Emilia, Modena, Italy; La Sapienza University of Rome, ITALY

## Abstract

The administration of painful primes has been shown to influence the perception of successively presented semantic stimuli. Painful primes lead to more negative valence ratings of pain-related, negative, and positive words than no prime. This effect was greater for pain-related than negative words. The identities of this effect’s neural correlates remain unknown. In this EEG experiment, 48 healthy subjects received noxious electrical stimuli of moderate intensity. During this priming, they were presented with adjectives of variable valence (pain-related, negative, positive, and neutral). The triggered event-related potentials were analyzed during N1 (120–180 ms), P2 (170–260 ms), P3 (300–350 ms), N400 (370–550 ms), and two late positive complex components (LPC1 [650–750 ms] and LPC2 [750–1000 ms]). Larger event-related potentials were found for negative and pain-related words compared to positive words in later components (N400, LPC1, and LPC2), mainly in the frontal regions. Early components (N1, P2) were less affected by the word category but were by the prime condition (N1 amplitude was smaller with than without painful stimulation, P2 amplitude was larger with than without painful stimulation). Later components (LPC1, LPC2) were not affected by the prime condition. An interaction effect involving prime and word category was found on the behavioral level but not the electrophysiological level. This finding indicates that the interaction effect does not directly translate from the behavioral to the electrophysiological level. Possible reasons for this discrepancy are discussed.

## Introduction

Numerous event-related potential (ERP) studies have shown that valence affects different stages of word processing (for reviews, see [1, 2]). Early stages up to 200 ms after the presentation of a written word are considered essential for identifying the visual word form and information about the word category, followed by semantic access at approximately 200 ms [2, 3]. Attentional resources are allocated at approximately 300 ms post-stimulus [4]. Contextual analysis and lexico-semantic retrieval occur at around 400 ms [5, 6], followed by continued processing, detailed word evaluation, and processes relating to memory encoding starting at approximately 500 ms post-stimulus [2].

The processing of written emotional words differs from that of neutral words. Most reported studies have used lexical decision, silent reading, or written word identification as tasks in their respective experimental paradigms. In most studies, the N1 peaks around 100–180 ms [1] and typically has a larger amplitude after the presentation of negative words than positive or neutral words. This difference was shown for the left anterior [7], centroparietal [8], and left posterior regions [7]. As a subcategory of negative words, pain-related words generate larger N1 amplitudes than neutral words in the left frontal regions [9]. Negative words elicit larger N1 amplitudes than positive words in the left temporal regions after priming with positive but not negative mood-inducing film clips [10]. Unlike these findings, Hofmann et al. described a processing advantage for negative and positive words compared to neutral words for the N1 [11]. Furthermore, larger amplitudes were found for positive words than negative and neutral words, although these effects were only trends [12]. Other studies [13, 14] found emotionally valenced words to have no effect on the N1.

According to Citron, the P2 usually peaks between 180 and 300 ms [1]. For this component, most studies found larger amplitudes for words with high emotional valence (negative and positive) than neutral words in the frontal [15] and centroparietal [14] regions or the whole scalp [16]. This finding is consistent with Kanske and Kotz [17], who found larger P2 amplitudes for positive than neutral words. However, other studies [18, 19] found emotionally valenced words to have no effect on the P2 amplitude.

Similar results can be found for the P3, which typically peaks around 300–400 ms [1], although very few studies have examined this component. Emotionally valenced words elicited larger P3 amplitudes than neutral words in the centroparietal [14] and parietal [20] regions, with the latter only tested for negative vs. neutral words. Hinojosa et al. [21] reported the opposite effect. In their study, neutral words elicited larger P3 amplitudes than emotionally valenced words (negative and positive) in several regions (frontopolar, middle frontal, right frontal, right parietal, and occipital). Several other studies found no valence effects for the P3 [8, 19, 22].

Mixed results are also found for the N400, which usually peaks around 390–590 ms [1]. Schacht and Sommer repeatedly reported larger N400 amplitudes after emotionally valenced words than neutral words in the frontotemporal [15] and occipitotemporal [23] regions. Unlike these findings, some studies suggest the opposite. Larger N400 amplitudes for neutral words have been reported in the frontocentral [16, 17, 22] and centroparietal [24] regions. However, larger N400 amplitudes for negative words than positive words were found in the centroparietal regions [13]. Kissler and Bromberek-Dyzman [10] could not show a valence effect on the N400 component.

Studies investigating the late positive complex (LPC), which usually peaks between 500 and 800 ms [1], are less ambiguous. Most studies found larger amplitudes for words with high emotional valence (negative and positive) than neutral words in the left frontal [16], central [18, 25], centroparietal [10, 25], parietal [15], and occipital [25] regions or the whole scalp [17]. Larger LPC amplitudes for positive than negative and neutral words were found in the central [12] and centroparietal [13, 14] regions. Interestingly, this effect was found for positive words in the centroparietal region only after priming with negative but not positive mood-inducing film clips [10]. In contrast, several studies show larger LPC amplitudes for negative than positive and neutral words in the central [7], centroparietal [11], and parietal [23] regions. Pain-related words elicit larger LPC amplitudes than neutral words in the frontal and central regions [9]. Another study found larger LPC amplitudes after pain-related vs. positive words only for chronic musculoskeletal pain patients, not for healthy controls [19].

While both early and late stages of word processing appear to be affected by the valence of words, results for early components such as N1, P2, or P3 are more ambiguous than for late components such as LPC. Some studies indicate an advantage in the processing of valenced over neutral words. Other studies have shown the opposite effect and, again, in some cases, negative words appear to have an advantage in processing over positive and neutral words. Late components more clearly indicate ongoing processing of emotionally valenced stimuli than neutral stimuli, although results here are also mixed. Our understanding of these processes is still incomplete, especially regarding the effects of pain-related words. Noxious primes can change the perceived valence of subsequently presented semantic stimuli [26]. Valence ratings of pain-related, negative, and positive–but not neutral–words became more negative after a painful electrical prime than no prime [26]. The question remains whether corresponding changes in the ERPs accompany these effects.

This study will determine whether pain-specific priming effects found in behavioral studies [26, 27] are reflected in the corresponding ERP components. This will broaden our knowledge of the interaction between acute pain and language processes as well as the corresponding neuronal mechanisms. Such knowledge could lead to the development of language-based interventions to modulate the perception of pain. Therefore, this study investigates the electrophysiological correlates of valenced words (negative and positive), focusing on pain-related words as a subcategory of negative words, compared to neutral words. Based on the previous research above, we hypothesize larger ERP amplitudes for emotionally valenced words than neutral words, especially for later components such as the N400 and LPC. Furthermore, Kissler and Bromberek-Dyzman [10] began to explore priming effects for valence-affected ERPs. According to their findings, we expect larger ERP amplitudes with emotionally valenced primes than no primes, especially for later components (N400 and LPC). Instead of using audiovisual primes, we investigate the effects of noxious primes on the ERPs of subsequently presented semantic stimuli, which has never been done before. Regarding the effects of painful stimuli on ERP responses, we did not have specific hypotheses. In our experiment, the interval between the onset of the painful primes and the analyzed ERPs varies because of the variable jitter between the onset of the painful prime and the onset of word presentation (this will be explained in more detail in the next section). As a consequence, it is difficult to make clear assumptions regarding the effects of painful stimuli on ERP responses in this experiment.

## Materials and methods

### Subjects

Forty-eight healthy right-handed native German-speaking students at the Friedrich Schiller University Jena (29 female and 19 male, aged 24.5 ± 4.0 years) participated in this experiment between November of 2018 and April of 2019. Written informed consent for participating according to the Declaration of Helsinki was obtained from all participants. This experiment was approved by the ethics committee of Friedrich Schiller University Jena (approval number: FSV 14/ 04) before commencement. We ensured that all subjects did not have acute or chronic pain, were not taking pain medication, and had no depressive symptoms. A Likert scaled (0–10) life pain questionnaire and a clinical interview combined with the Beck Depression Inventory 2 [28] were used. Subjects received a monetary reimbursement of 8.50 Euros per hour. After the participation in the experiment no information remained that could identify individual participants.

### Stimulus materials and procedure

The materials and procedure were the same as in our previous study [26]. We used monophasic electrical stimuli of 500 μs with a frequency of 200 Hz as primes with a duration of 3.5 s. They were generated by a constant current stimulator (DS7H; Digitimer, Welwyn Garden City, UK). The intracutaneous stimulation method was used to apply these stimuli to the tip of the middle finger on the left hand [29–31]. Skin resistance was reduced by inserting an isolated golden pin electrode (diameter: 0.95 mm, length: 1 mm) into a small epidermal cavity, 1 mm in diameter and about 1 mm in depth, and fixed with adhesive tape. Care was taken not to cause any bleeding. A flexible stainless-steel electrode, fixed loosely around the first finger joint of the middle finger, served as the reference electrode.

The participants’ pain sensitivity was determined by giving them electrical stimuli according to the following procedure. The subjects received an electrical stimulus and were asked to rate it after it had stopped. The stimulation intensity was changed according to the subject’s rating. We used the method of limits to determine perception thresholds for somatosensory sensation and intensity to evoke moderately painful perceptions. Subjects were asked to rate each stimulus on a modified Ellermeier scale [32, 33] comprising eight verbal categories. Each category was scored as follows: 0, no sensation; 1–10, just perceived but not painful; 11–20, clearly perceived but not painful; 21–30, very mildly painful; 31–40, mildly painful; 41–50, moderately painful; 51–60, strongly painful; 61–70, very strongly painful; see [33] for a more detailed description of the scale. The scale was presented on the same computer screen where the words and the valence scale were presented later in the experiment. The subjects were prompted to give their numerical rating verbally. The stimulation started at 0 mA and gradually increased in 0.05 mA steps until the subject reported a first sensation (i.e., their rating was >0 for the first time). Then, the stimulation decreased by 0.05 mA until the subjects gave a pain rating of 0 again. Such increases and decreases were repeated four times around the threshold for a reported sensation. The last three intensities were used to determine the threshold. After determining the somatosensory perception threshold, the intensity was increased in 0.5 mA steps until the second threshold (fixed at a rating of 50), indicating the boundary of moderate to strongly painful pain, was determined. We used a rating of 50 since we expected some habituation during the experiment. We applied the same procedure as for the first threshold (increasing and decreasing intensity four times). The intensity threshold identified this way was used as the electrical stimulus in the experiment. A moderate pain stimulus was used because it has been shown that priming effects with electrical stimulation only occur for clearly painful and not for lower-intensity stimulation slightly above the pain threshold [31, 34, 35].

Similar to previous studies, we used 40 German adjectives as target stimuli in four categories: 10 pain-related (e.g., excruciating), 10 negative (e.g., hostile), 10 neutral (e.g., cubical), and 10 positive adjectives (e.g., exhilarating) [31, 35]. Between categories, words were matched for their word frequency and length. Valence and arousal qualities were balanced for pain-related and negative words. Furthermore, arousal qualities of pain-related, negative, and positive words were balanced (see [35] for a more detailed description of the method). Adjectives were shown in a pseudo-randomized order. We ensured that no more than two consecutive words were from the same category to prevent summation effects.

**Fig 1:**
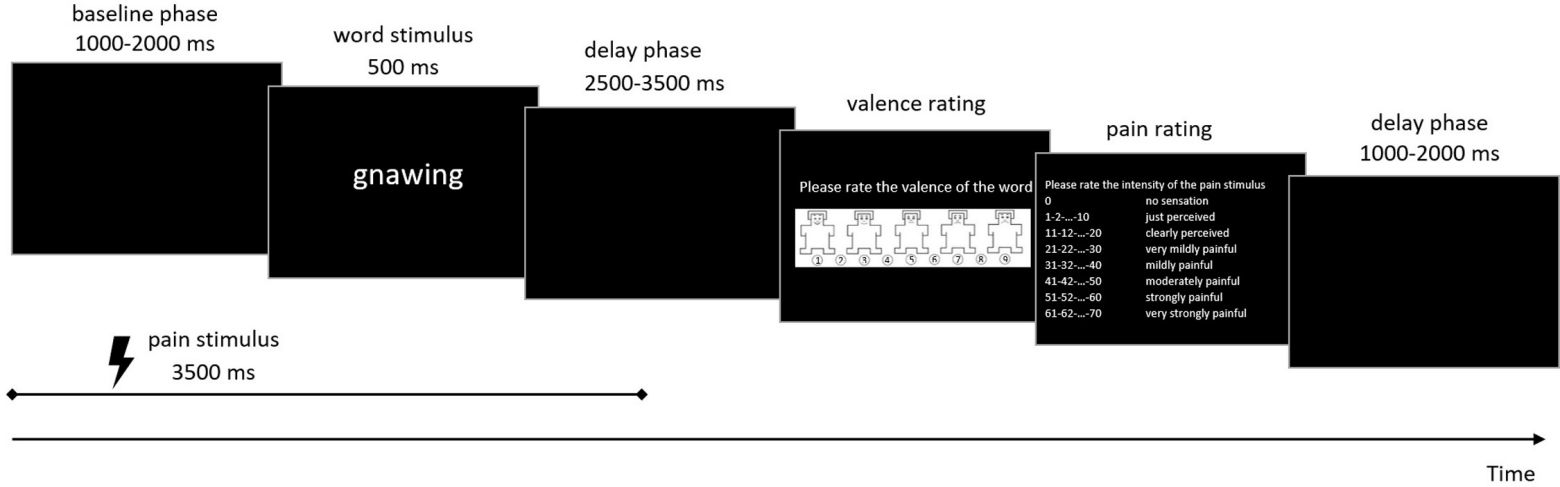
Design of the experimental trials with painful stimulation. The adjective used in the figure and the scale were translated from German to English for illustration. In control trials, the timing was the same but without painful stimulation.

The experiment was divided into four blocks of 100 trials, each lasting 14 minutes. During the one-minute rest interval between blocks, subjects could drink a glass of water or relax. The experiment was performed in a special experimental noise-attenuating cabin (Industrial Acoustics Company GmbH, Niederkrüchten, Germany) and took approximately two hours. In each trial (Fig 1), an adjective was presented on the screen for 500 ms for the participants to read. The offset of the adjective presentation was followed by 2.5–3.5 s of black screen (jitter), which was followed by the presentation of the valence scale (Self-Assessment Manikin: 1 [most positive] to 9 [most negative]) [36] to assess the valence of the shown word. The subjects gave their answer verbally. All subjects’ ratings were entered on the keyboard by the experimenter. Additionally, on average, participants were shown the modified Ellermeier scale to rate the pain sensation verbally every seventh trial. At the end of the trial, there was a black screen for 1–2 s (jitter) before the next trial started.

The trial’s primary structure was modified by the presence or absence of an antecedent painful stimulation. Half of the trials did not have painful electrical stimulation before presenting the adjective, while the other half did. In the latter condition, the trial was as described above but with additional electrical stimulation. Painful electrical stimulation started at the beginning of the trial before an adjective was presented. An adjective was presented for 500 ms 1–2 s (first jitter) after the onset of the electrical stimulation. The electrical stimulation lasted throughout the presentation of the adjective. The presentation of the scales was as described above. In total, 200 trials with and 200 without painful electrical stimulation were conducted. The order of trials with and without painful electrical stimulation was randomized to avoid the effects of expectations (see [37] for those effects). Therefore, each word was presented five times with and five times without painful electrical stimulation. Jitters were randomized and were applied to prevent a precise prediction of the onset of the next stimulus. The experiment was controlled by the Presentation software (Version 14.5, Neurobehavioral Systems, Inc., Albany, CA, USA).

### EEG recording

A continuous electroencephalogram (EEG) was recorded using 64 Ag-AgCl electrodes. They were placed on the scalp according to the extended international 10–20 system [38, 39]. The electrode impedances were kept <5 kΩ. The FCz electrode was used as the online reference. The electrooculogram (EOG) was recorded from an electrode placed below the lower eyelid of the left eye. EEG and EOG data were amplified by two Neuroscan SynAmps (Herndon, VA, USA) and recorded with Brain Vision Recorder software (Brain Products GmbH, Munich, Germany). Data was bandpass filtered (0.05–500 Hz), then digitized at 2000 Hz (with a resolution of 16-bit), and stored offline on a hard disk.

### Data analysis

The EEG data were preprocessed using Vision Analyzer 2 software (Brain Vision, Munich, Germany). Portions of the data contaminated by ocular movements or eye blinks were corrected according to the procedure by Gratton et al. [40]. Data were bandpass filtered between 1 and 30 Hz, and segmentation was performed in the time window from 200 ms before to 1500 ms after the presentation of the word. After visual inspection, the remaining artifacts were removed. The baseline correction used the prestimulus interval from −500 to 0 ms before the presentation of the word. Based on visual inspection of the grand averages and previous findings mentioned above, we identified six distinct ERP components: N1 (120–180 ms), P2 (170–260 ms), P3 (300–350 ms), N400 (370–550 ms), and two LPC components: LPC1 (650–750 ms) and LPC2 (750–1000 ms). Based on these time windows, the peak amplitudes for each component were exported.

**Fig 2:**
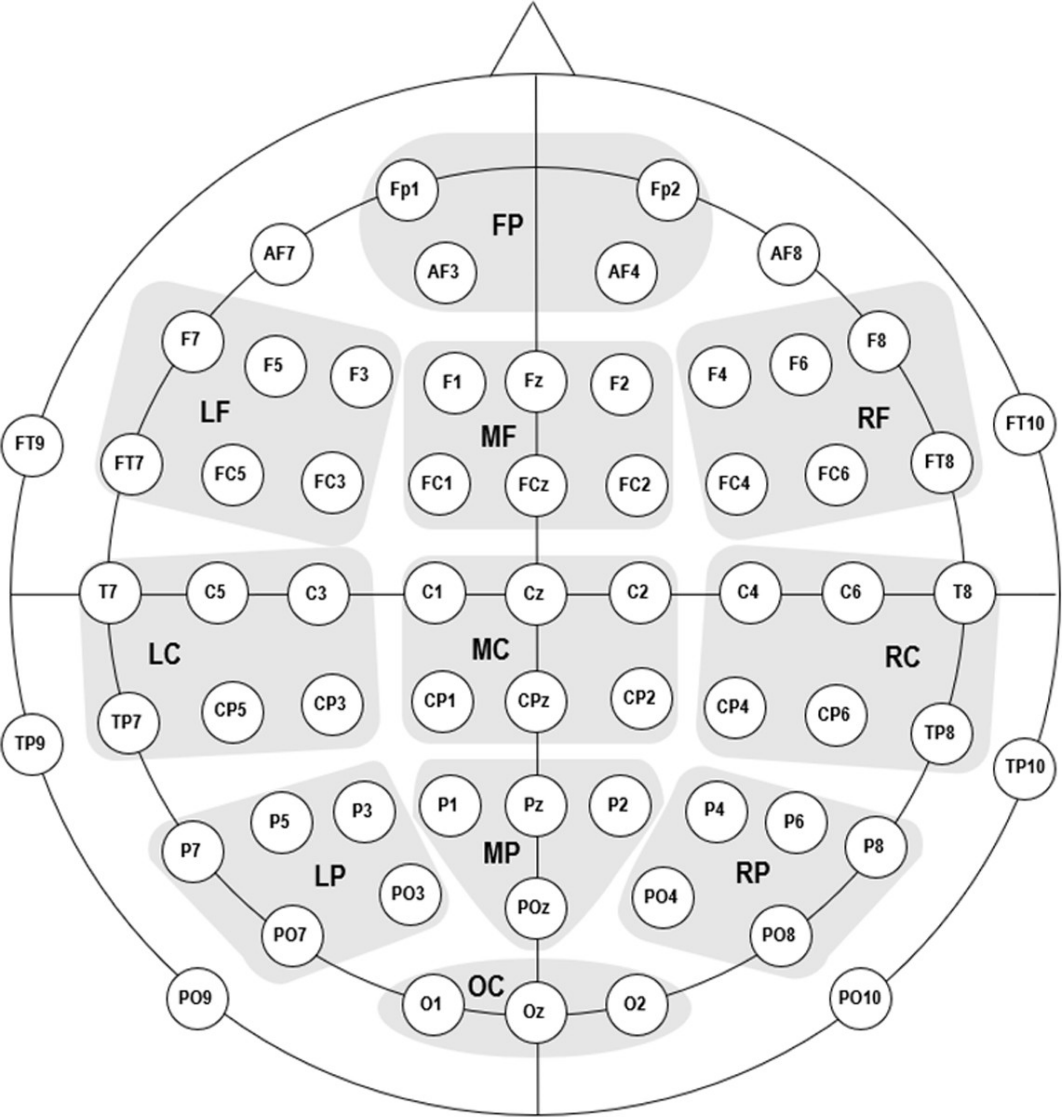
Grouping of regions for the analysis of ERPs. FP = frontopolar, LF = left frontal, MF = middle frontal, RF = right frontal, LC = left central, MC = middle central, RC = right central, LP = left parietal, MP = middle parietal, RP = right parietal, OC = occipital.

The preprocessed EEG data were then analyzed with the Statistical Package for the Social Sciences software (version 22.0, IBM Corp., Armonk, NY, USA). Individual electrodes were clustered into 11 regions of interest as described by Hinojosa [25] (Fig 2). The activities of all associated electrodes were averaged within each region. Analyses were also conducted with only four regions of interest (according to Pauligk et al. [16] or Kanske and Kotz [17]). Since the results did not vary, we only present those for the 11 regions of interest.

The effects of painful primes were assessed using repeated measures analyses of variance (ANOVA) with the *Category* (pain-related, negative, neutral, and positive adjectives), *Prime* (stimulation vs. no stimulation), and *Region* (see Fig 2) as within-subject factors for each ERP component. Only within-subject factors *Category* and *Prime* were used for the behavioral data. Degrees of freedom were corrected using the Greenhouse–Geisser procedure to account for violations of sphericity [41]. Interactions were then analyzed using simple effect analyses. Significant main effects were followed by post hoc paired-sample t-tests (two-tailed, Bonferroni–Holm corrected). We report η_p_^2^ as a measure of effect size [42].

## Results

### Behavioral data

**Fig 3:**
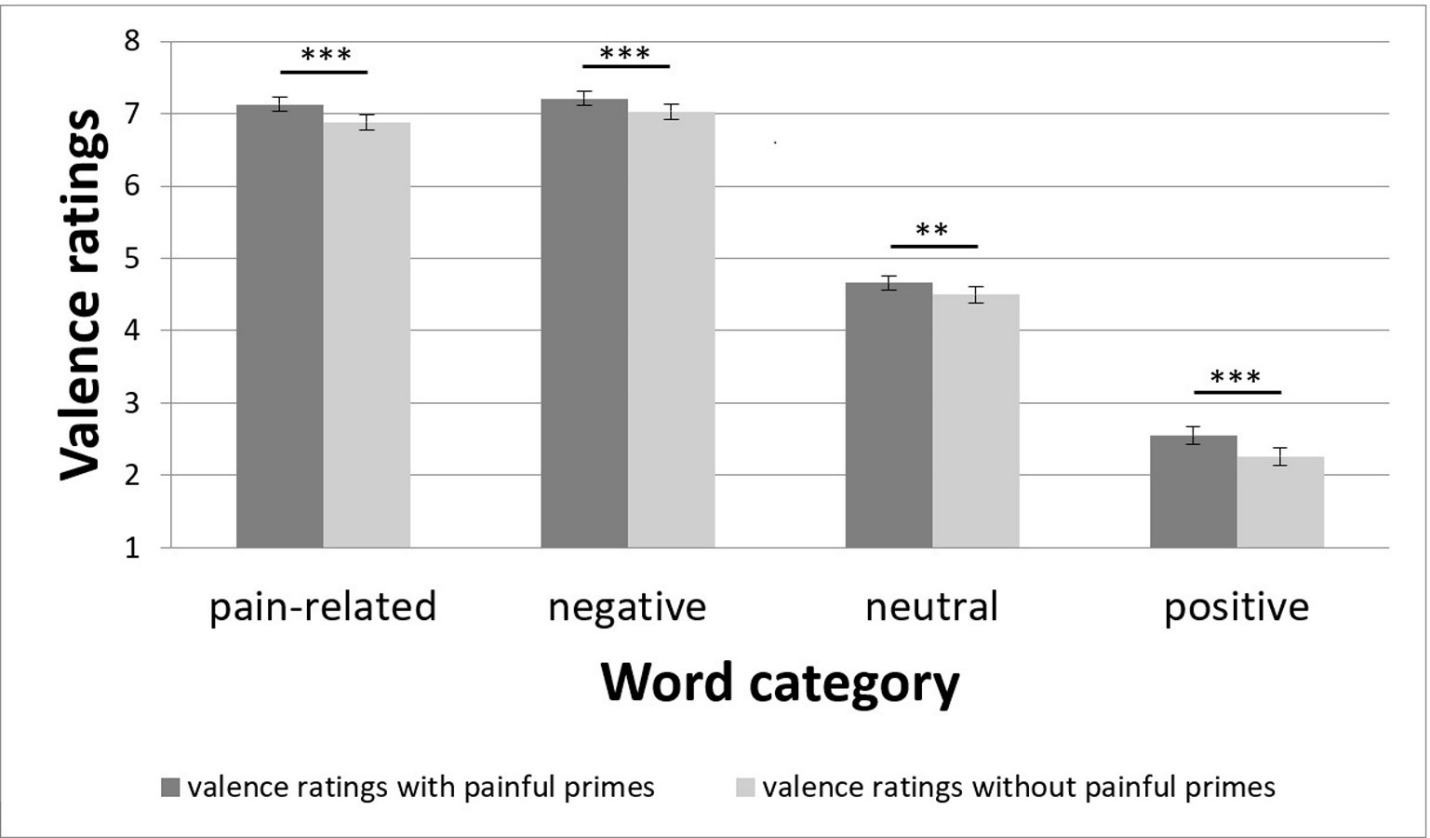
Valence ratings for the factors prime and category. Data are shown as mean (SE); ***: p < 0.001, **: p < 0.01.

For valence ratings, ANOVA indicated significant main effects for *Prime* (F_(1; 47)_ = 19.41; p < 0.001; η_p_^2^ = 0.29) and *Category* (F_(1.8; 84.8)_ = 528.51; p < 0.001; η_p_^2^ = 0.92) and a significant interaction for Prime*Category (F_(2.1; 99.5)_ = 5.03; p = 0.007; η_p_^2^ = 0.10). Since the *Prime* factor only has two levels, the main effect showed a significant prime vs. no prime effect, with more negative valence ratings (mean ± standard error [SE]) with (5.39 ± 0.06) than without (5.17 ± 0.06) a painful prime. Regarding the main effect of *Category*, t-tests between its levels indicated highly significant differences for all comparisons except for pain-related vs. negative words (t = −1.71; p = 0.094). The other comparisons for the main effect of *Category* (the category with more negative valence ratings is given first): pain-related vs. neutral words (t = 19.81; p < 0.001), pain-related vs. positive words (t = 26.19; p < 0.001), negative vs. neutral words (t = 20.77; p < 0.001), negative vs. positive words (t = 26.52; p < 0.001), and neutral vs. positive words (t = 16.74; p < 0.001). The interaction effect for Prime*Category indicated significantly higher valence ratings with painful vs. no stimulation for pain-related (t = 5.61; p < 0.001), negative (t = 4.65; p < 0.001), neutral (t = 2.75; p = 0.008), and positive (t = 3.97; p < 0.001) words (Fig 3). The difference in valence ratings between painful vs. no stimulation was largest for positive words (2.55 ± 0.12 with vs. 2.26 ± 0.12 without a painful prime), followed by pain-related (7.13 ± 0.10 vs. 6.88 ± 0.10), negative (7.21 ± 0.10 vs. 7.03 ± 0.11), and neutral (4.66 ± 0.10 vs. 4.50 ± 0.11) words.

### Electrophysiological data

#### N1

ANOVA indicated two significant main effects for *Region* (F_(2.5; 116.7)_ = 27.35; p < 0.001; η_p_^2^ = 0.37) and *Prime* (F_(1; 47)_ = 22.81; p < 0.001; η_p_^2^ = 0.33) and a significant interaction for Region*Prime (F_(3.8; 179.9)_ = 4.54; p = 0.002; η_p_^2^ = 0.09).

Overall, the N1 amplitude was smaller with (0.03 ± 0.02 μV) than without (−0.03 ± 0.03 μV) a painful prime (t = 4.78; p < 0.001). Post hoc t-tests for the main effect of *Region* showed negative amplitudes for frontocentral regions and positive amplitudes for parietooccipital regions. For the detailed results of the contrasts for *Region*, see S1 Table in the Supporting Information.

**Fig 4:**
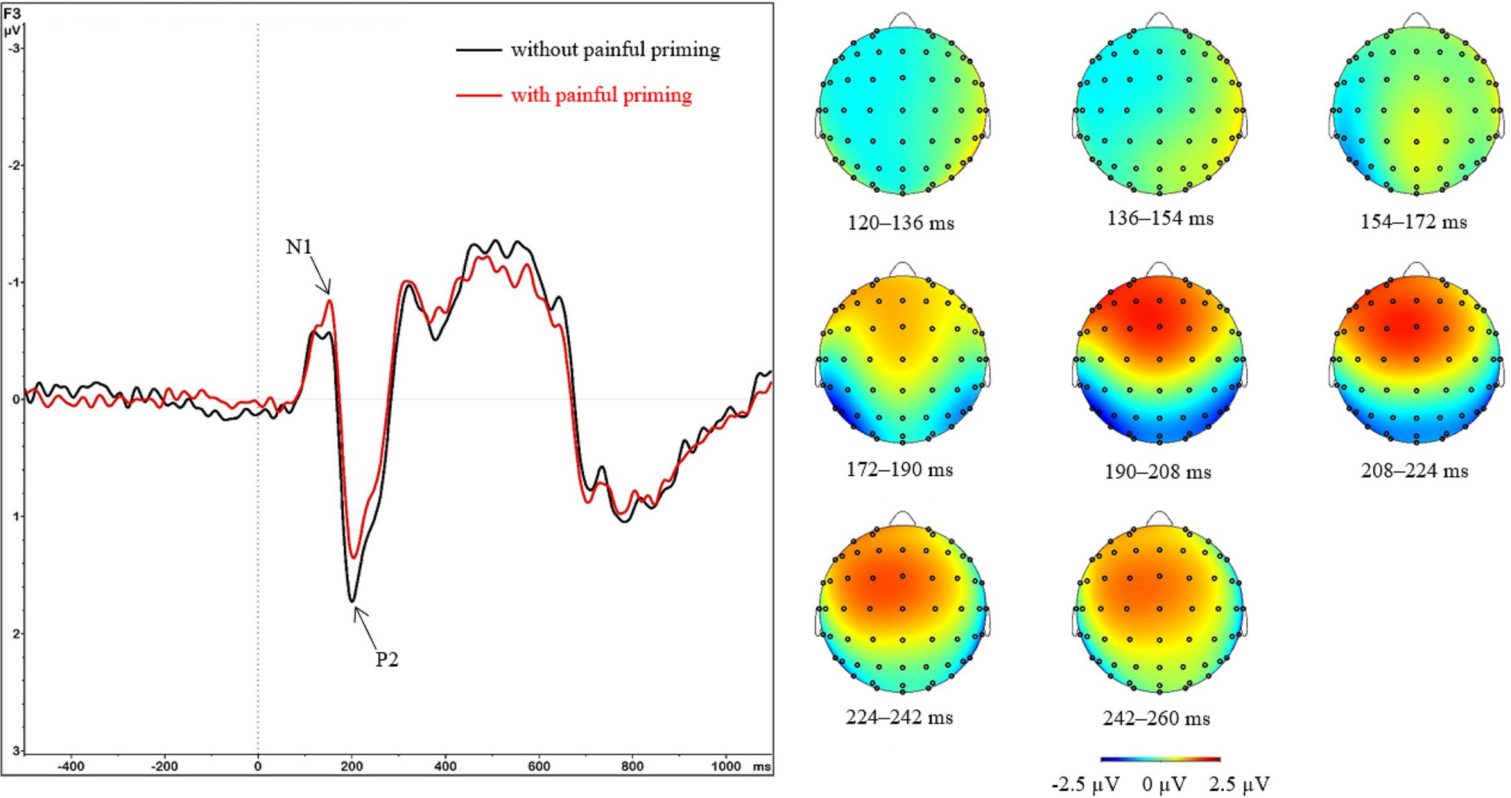
Grand averages and topographic maps for early potentials. Left: Grand average ERPs from left frontal electrode F3 with vs. without painful priming. Right: Topographic maps of the time intervals of N1 and P2 for trials without painful priming.

**Fig 5:**
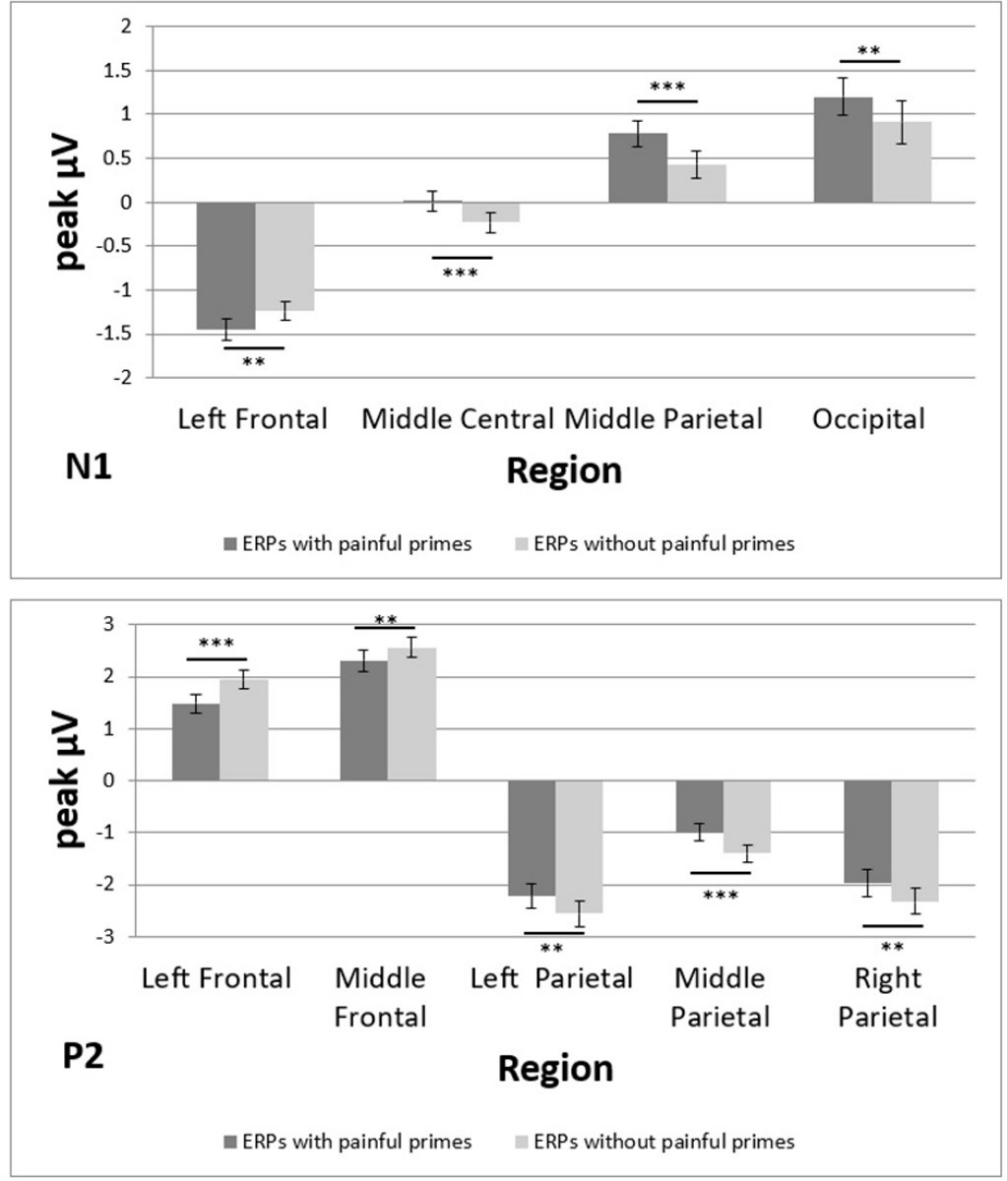
ERPs for different regions (top: N1, bottom: P2). Data are shown as mean μV (SE); ***: p < 0.001, **: p < 0.01, *: p < 0.05.

The interaction effect of Region*Prime was followed by simple effect analyses of the factor *Prime* for each level of the *Region* factor. Significant simple effects were found for the left frontal (F_(1; 47)_ = 7.50; p = 0.009; η_p_^2^ = 0.14), middle central (F_(1; 47)_ = 14.68; p < 0.001; η_p_^2^ = 0.24), middle parietal (F_(1; 47)_ = 18.04; p < 0.001; η_p_^2^ = 0.28), and occipital (F_(1; 47)_ = 7.37; p = 0.009; η_p_^2^ = 0.14) regions (Fig 4). Since the *Prime* factor only has two levels, the simple effects show a significant stimulation vs. no stimulation effect. In the left frontal region, the N1 amplitude was larger with (−1.45 ± 0.13 μV) than without (−1.24 ± 0.11 μV) a painful prime. In all other regions, it was the opposite (with a painful prime given first): middle central (0.01 ± 0.11 μV vs. −0.23 ± 0.11 μV), middle parietal (0.78 ± 0.14 μV vs. 0.43 ± 0.15 μV), occipital (1.20 ± 0.21 μV vs. 0.91 ± 0.25 μV; Fig 5).

#### P2

Similar to the N1, ANOVA indicated two significant main effects for *Region* (F_(2.2; 102.4)_ = 88.81; p < 0.001; η_p_^2^ = 0.65) and *Prime* (F_(1; 47)_ = 17.90; p < 0.001; η_p_^2^ = 0.28) and a significant interaction for Region*Prime (F_(3.5; 164.2)_ = 7.95; p < 0.001; η_p_^2^ = 0.15).

Overall, the P2 amplitude was larger with (−0.02 ± 0.03 μV) than without (−0.07 ± 0.03 μV) a painful prime (t = 4.23; p < 0.001). Post hoc t-tests for the main effect of *Region* showed positive amplitudes for frontal regions and negative amplitudes for parietooccipital regions. For the detailed results of the comparisons for *Region*, see S1 Table in the Supporting Information.

Regarding the interaction of Region*Prime, simple effect analyses of the *Prime* factor were conducted for each level of the *Region* factor. Significant simple effects were found for the left frontal (F_(1; 47)_ = 19.19; p < 0.001; η_p_^2^ = 0.29), middle frontal (F_(1; 47)_ = 8.58; p = 0.005; η_p_^2^ = 0.15), left parietal (F_(1; 47)_ = 9.29; p = 0.004; η_p_^2^ = 0.17), middle parietal (F_(1; 47)_ = 14.17; p < 0.001; η_p_^2^ = 0.23), and right parietal (F_(1; 47)_ = 10.41; p = 0.002; η_p_^2^ = 0.18) regions (Fig 4). Since the *Prime* factor only has two levels, the simple effects show a significant stimulation vs. no stimulation effect. In the left and middle frontal regions, the activation was less positive with (1.48 ± 0.18 and 2.31 ± 0.20 μV, respectively) than without (1.94 ± 0.18 and 2.55 ± 0.19 μV, respectively) a painful prime. In the other regions, it was the opposite (with a painful prime given first): left parietal (−2.21 ± 0.24 μV vs. −2.56 ± 0.25 μV), middle parietal (−0.99 ± 0.16 μV vs. −1.40 ± 0.18 μV), right parietal (−1.98 ± 0.26 μV vs. −2.33 ± 0.24 μV; Fig 5).

#### P3

Only the main effect for *Region* (F_(2.9; 137.9)_ = 86.89; p < 0.001; η_p_^2^ = 0.65) achieved statistical significance. None of the interactions were significant. Post hoc t-tests indicated negative amplitudes for frontal regions and positive amplitudes for parietooccipital regions. For the detailed results of the comparisons for *Region*, see S1 Table in the Supporting Information.

#### N400

ANOVA indicated a significant main effect for *Region* (F_(2.9; 137.3)_ = 172.68; p < 0.001; η_p_^2^ = 0.79) and two significant interactions of Region*Category (F_(8.4; 396.4)_ = 3.37; p < 0.001; η_p_^2^ = 0.07) and Region*Prime (F_(3.3; 154.6)_ = 3.67; p = 0.011; η_p_^2^ = 0.07).

Post hoc t-tests for the main effect of *Region* showed positive amplitudes for frontal regions and negative amplitudes for parietooccipital regions. For the detailed results of the comparisons for *Region*, see S1 Table in the Supporting Information.

**Fig 6:**
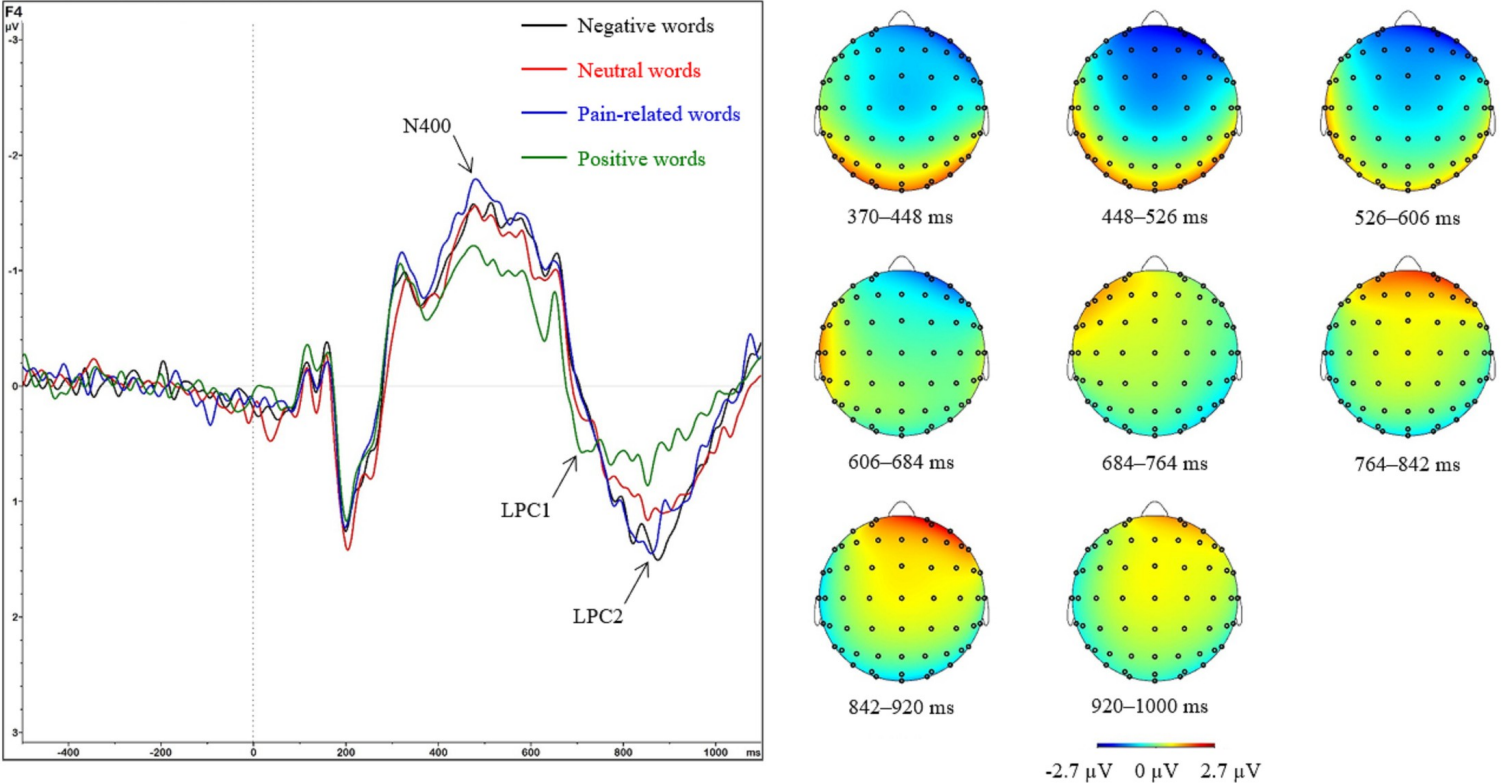
Grand averages and topographic maps for late potentials. Left: Grand average ERPs from the right frontal electrode F4 for all word categories. Right: Topographic maps of the time intervals of N400, LPC1, and LPC2 for trials with pain-related words. Note the activity shift from left to right frontal regions between LPC1 and LPC2.

**Fig 7:**
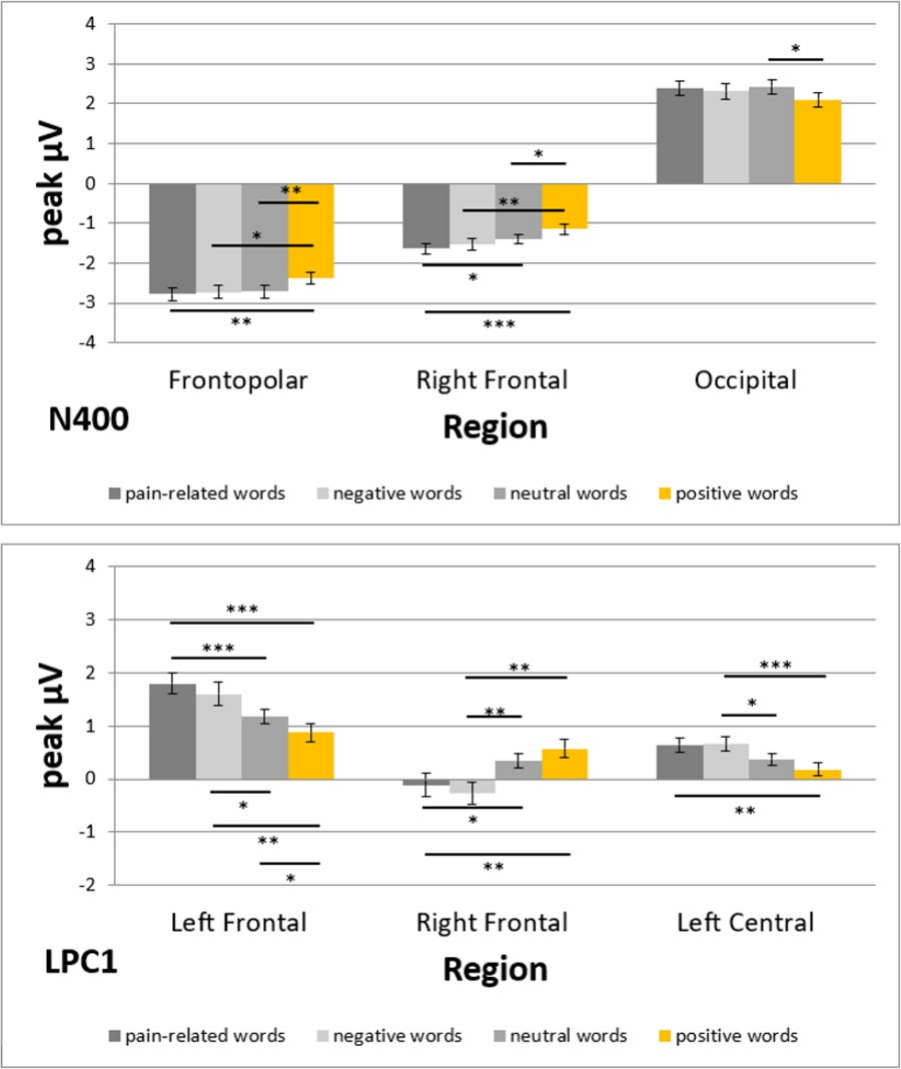
ERPs for the factors Region and Category (top: N400, bottom: LPC1). Data are shown as mean μV (SE); ***: p < 0.001, **: p < 0.01, *: p < 0.05.

The interaction effect of Region*Category was followed by simple effect analyses of the *Category* factor for each level of the *Region* factor. Significant simple effects were found for the frontopolar (F_(3; 45)_ = 5.32; p = 0.003; η_p_^2^ = 0.26), right frontal (F_(3; 45)_ = 5.71; p = 0.002; η_p_^2^ = 0.28), right parietal (F_(3; 45)_ = 3.14; p = 0.034; η_p_^2^ = 0.17), and occipital (F_(3; 45)_ = 3.67; p = 0.019; η_p_^2^ = 0.20) regions (Fig 6). The differences between categories were assessed using separate post hoc t-tests for the respective regions. In the frontopolar region, significant differences were found for pain-related vs. positive (t = −3.67; p = 0.003), negative vs. positive (t = −3.18; p = 0.010), and neutral vs. positive (t = −3.77; p = 0.003) words, with pain-related (−2.78 ± 0.16 μV), negative (−2.72 ± 0.17 μV), and neutral (−2.71 ± 0.16 μV) words all more negative than positive words (−2.38 ± 0.16 μV). In the right frontal region, significant differences were found for pain-related vs. neutral (t = −2.51; p = 0.047), pain-related vs. positive (t = −4.11; p < 0.001), negative vs. positive (t = −3.35; p = 0.008), and neutral vs. positive (t = −2.64; p = 0.045) words, with pain-related (−1.64 ± 0.13 μV), negative (−1.52 ± 0.14 μV), and neutral (−1.40 ± 0.12 μV) words all more negative than positive words (−1.15 ± 0.12 μV). In the right parietal region, none of the post hoc t-tests were significant after correction, although the difference between pain-related vs. positive words was marginally significant (t = 2.72; p = 0.054). Finally, in the occipital region, a significant difference was observed between neutral vs. positive words (t = 3.21; p = 0.014), with neutral words (2.43 ± 0.17 μV) being more positive than positive words (2.10 ± 0.17 μV; Fig 7).

Regarding the interaction of Region*Prime, simple effect analyses of the *Prime* factor were conducted for each level of the *Region* factor. Significant simple effects were found for the right frontal (F_(1; 47)_ = 8.95; p = 0.004; η_p_^2^ = 0.16), right central (F_(1; 47)_ = 8.94; p = 0.004; η_p_^2^ = 0.16), and occipital (F_(1; 47)_ = 6.75; p = 0.012; η_p_^2^ = 0.13) regions. Since the *Prime* factor only has two levels, the simple effects show a significant stimulation vs. no stimulation effect. In the right frontal and right central regions, the activation was more negative with (−1.58 ± 0.13 and 0.20 ± 0.10 μV, respectively) than without (−1.28 ± 0.12 and 0.46 ± 0.09 μV, respectively) a painful prime. In the occipital region, it was the opposite: 2.45 ± 0.18 μV with and 2.14 ± 0.15 μV without a painful prime.

#### LPC1

ANOVA indicated a significant main effect for *Region* (F_(3; 141.4)_ = 42.61; p < 0.001; η_p_^2^ = 0.48) and *Category* (F_(2.5; 117.1)_ = 5.21; p = 0.004; η_p_^2^ = 0.10) and a significant interaction of Region*Category (F_(6.4; 302.7)_ = 5.17; p < 0.001; η_p_^2^ = 0.10).

Regarding the main effect for *Category*, post hoc t-tests indicated larger LPC1 amplitudes for positive words (−0.03 ± 0.02 μV) than for pain-related (−0.10 ± 0.02 μV; t = 3.84; p = 0.002) and negative (−0.09 ± 0.02 μV; t = 3.36; p = 0.008) words. The other comparisons were not significant, including those for neutral words (−0.07 ± 0.02 μV): pain-related vs. negative (t = −0.64; p = 0.835), pain-related vs. neutral (t = −1.28; p = 0.624), negative vs. neutral (t = −0.82; p = 0.835), and neutral vs. positive (t = −2.30; p = 0.104) words. Post hoc t-tests for the main effect of *Region* showed positive amplitudes for frontocentral regions and negative amplitudes for parietooccipital regions. For the detailed results of the contrasts for *Region*, see S1 Table in the Supporting Information.

The interaction effect of Region*Category was followed by simple effect analyses of the *Category* factor for each level of the *Region* factor. Significant simple effects were found for the frontopolar (F_(3; 45)_ = 3.51; p = 0.023; η_p_^2^ = 0.19), left frontal (F_(3; 45)_ = 7.71; p < 0.001; η_p_^2^ = 0.34), right frontal (F_(3; 45)_ = 5.11; p = 0.004; η_p_^2^ = 0.25), and left central (F_(3; 45)_ = 6.01; p = 0.002; η_p_^2^ = 0.29) regions (Fig 6). The differences between categories were assessed using separate post hoc t-tests for the respective regions. In the frontopolar region, none of the post hoc t-tests were significant after correction. In the left frontal region, significant differences were found for pain-related vs. neutral (t = 4.51; p < 0.001), pain-related vs. positive (t = 4.75; p < 0.001), negative vs. neutral (t = 2.71; p = 0.028), negative vs. positive (t = 3.60; p = 0.003), and neutral vs. positive (t = 2.61; p = 0.028) words, with pain-related (1.80 ± 0.19 μV) and negative (1.60 ± 0.22 μV) words having larger LPC1 amplitudes than neutral words (1.17 ± 0.14 μV), which had larger amplitudes than positive words (0.88 ± 0.17 μV). In the right frontal region, significant differences were found for pain-related vs. neutral (t = −2.92; p = 0.016), pain-related vs. positive (t = −3.25; p = 0.009), negative vs. neutral (t = −3.67; p = 0.003), and negative vs. positive (t = −3.85; p = 0.002) words, with neutral (0.34 ± 0.13 μV) and positive (0.57 ± 0.17 μV) words having larger LPC1 amplitudes than pain-related (−0.11 ± 0.21 μV) and negative (−0.26 ± 0.21 μV) words. Finally, in the left central region, significant differences were found for pain-related vs. positive (t = 3.43; p = 0.006), negative vs. neutral (t = 3.03; p = 0.016), and negative vs. positive (t = 4.22; p < 0.001) words, with pain-related (0.64 ± 0.13 μV) and negative (0.66 ± 0.14 μV) words having larger LPC1 amplitudes than neutral (0.37 ± 0.10 μV) and positive (0.18 ± 0.13 μV) words (Fig 7).

#### LPC2

ANOVA indicated a significant main effect for *Region* (F_(2.4; 111.7)_ = 81.78; p < 0.001; η_p_^2^ = 0.64) and a significant interaction of Region*Category (F_(8.5; 400.8)_ = 4.34; p < 0.001; η_p_^2^ = 0.09).

Post hoc t-tests for the main effect of *Region* showed positive amplitudes for frontal regions and negative amplitudes for parietooccipital regions. For the detailed results of the comparisons for *Region*, see S1 Table in the Supporting Information.

The interaction effect of Region*Category was followed by simple effect analyses of the *Category* factor for each level of the *Region* factor. Significant simple effects were found for the frontopolar (F_(3; 45)_ = 3.41; p = 0.025; η_p_^2^ = 0.19), left frontal (F_(3; 45)_ = 6.12; p = 0.001; η_p_^2^ = 0.29), right frontal (F_(3; 45)_ = 9.14; p < 0.001; η_p_^2^ = 0.38), left central (F_(3; 45)_ = 3.52; p = 0.022; η_p_^2^ = 0.19), and right central (F_(3; 45)_ = 4.01; p = 0.013; η_p_^2^ = 0.21) regions (Fig 6). The differences between categories were assessed using separate post hoc t-tests for the respective regions. In the frontopolar region, a significant difference was only found for pain-related vs. positive words (t = 2.84; p = 0.040), with pain-related words (3.04 ± 0.30 μV) having a larger LPC2 amplitude than positive words (2.53 ± 0.26 μV). In the left frontal region, significant differences were found for pain-related vs. positive (t = −2.83; p = 0.027), negative vs. neutral (t = −3.12; p = 0.015), and negative vs. positive (t = −4.26; p < 0.001) words, with neutral (0.44 ± 0.23 μV) and positive (0.65 ± 0.22 μV) words having larger LPC2 amplitudes than pain-related (0.15 ± 0.27 μV) and negative (−0.04 ± 0.27 μV) words. In the right frontal region, significant differences were found for pain-related vs. positive (t = 4.79; p < 0.001), negative vs. positive (t = 4.32; p < 0.001), and neutral vs. positive (t = 3.67; p = 0.003) words, with pain-related (2.04 ± 0.24 μV), negative (2.01 ± 0.25 μV), and neutral (1.69 ± 0.15 μV) words all more positive than positive words (1.22 ± 0.13 μV). In the left central region, significant differences were found for pain-related vs. positive (t = −2.90; p = 0.028) and negative vs. positive (t = −3.08; p = 0.021) words, with pain-related (−1.06 ± 0.15 μV) and negative (−1.05 ± 0.14 μV) words more negative than neutral (−0.87 ± 0.11 μV) and positive (−0.69 ± 0.09 μV) words. Finally, in the right central region, a significant difference was only found for negative vs. positive words (t = 3.41; p = 0.008), with pain-related (−0.07 ± 0.10 μV), negative (−0.02 ± 0.12 μV), and neutral (−0.17 ± 0.12 μV) words more positive than positive words (−0.29 ± 0.12 μV).

## Discussion

This study investigated valence ratings and their electrophysiological correlates to different word categories depending on painful primes. At the behavioral level, as expected, we found more negative valence ratings after painful stimulation vs. no stimulation. This effect was greatest for positive words, followed by pain-related, negative, and neutral words. Our major findings for the electrophysiological correlates are larger ERP amplitudes for negative and pain-related words than positive words in later components (N400, LPC1, and LPC2), mostly in the frontal regions. This finding partly confirms our hypothesis that later components, such as the N400 and LPC, would show larger ERP amplitudes for emotionally valenced words. Early components, such as N1, P2, and P3, appear unaffected by word category but affected by the prime.

### N400

We found larger N400 amplitudes for pain-related, negative, and neutral words than positive words in the right frontal regions. In addition, the N400 was larger for pain-related than for neutral words. This finding is consistent with other studies that found larger N400 amplitudes for neutral words than positive words at anterior electrode sites [16, 22]. Furthermore, it is consistent with Schacht and Sommer, who repeatedly reported larger N400 amplitudes after emotionally valenced words than neutral words [15, 23]. Most importantly, larger N400 amplitudes for negative words than positive words with a right dominant focus were also found by others [13, 43, 44]. It has been repeatedly suggested that later ERP components reflect emotional processing and therefore indicate higher-order evaluation processes [17, 45, 46]. It has also been repeatedly suggested that the N400 plays a role in contextual analysis and lexico-semantic retrieval [5, 6]. It appears that positive words trigger less electrophysiological activity in the time frame of the N400, leading their lexico-semantic retrieval to be more facilitated than pain-related, negative, and neutral words. This processing advantage of positive words during N400 can be explained by a general mildly positive mood in healthy subjects [47]. Such a positive mood bias could have facilitated the processing of positive words.

### LPC

We found larger LPC1 amplitudes for pain-related and negative words than for neutral and positive words in the left frontocentral regions. The results were reversed for the right frontocentral region, where we found larger amplitudes for neutral and positive words than for pain-related and negative words. Furthermore, we found the opposite pattern for the LPC2. Here, neutral and positive words generated larger amplitudes than pain-related and negative words in the left frontocentral regions. In contrast, pain-related and negative words generated larger amplitudes than neutral and positive words in the right frontocentral regions.

Our results for the LPC1 are partly consistent with various studies mentioned in the introduction [10, 16–18, 25] and partly contradict others [12, 13]. Our results for the LPC2 are also partly consistent with some studies mentioned in the introduction [12, 13] and partly contradict others [10, 16–18, 25]. The contradictory results for the LPC might be due to the different time frames in which the LPC was investigated, the different word classes used (adjectives, nouns, and verbs), or the different tasks used (lexical decision, silent reading, or written word identification). However, we found no systematic influences of these factors when examining the other studies. While some studies have shown larger amplitudes for positive words than negative words for early LPC time frames, such as 550–650 ms after word onset [12, 13], others have shown the opposite effect in the same LPC time frame [11, 23]. This inconsistency also exists for the different word classes. While some studies have shown larger LPC amplitudes for positive adjectives than negative adjectives [10, 14], others using adjectives have shown the opposite effect [7, 19]. Therefore, there must be another explanation for the heterogeneous results regarding the LPC.

Using two consecutive LPC time frames, we found an activity shift in frontal regions between LPC1 and LPC2. This shift might explain the contradictory results of other studies highlighted above. LPC1 amplitudes were larger for pain-related and negative words than for neutral and positive words in the left frontocentral regions. However, the opposite was observed in the right frontocentral region. This frontocentral activity pattern in LPC1 then shifts to the contralateral regions in LPC2. Few studies have investigated ERPs’ lateralization for valenced stimuli. Emotion effects appear larger in the right regions [48, 49]. Activity in the left regions might refer to language-related processes [13, 50].

In the temporal dimension, LPC effects are assumed to indicate continued processing, detailed word evaluation, and processes related to memory encoding [1, 2]. The activity patterns could not be explained by arousal of the word, word length, or word frequency because we controlled for these characteristics. However, during LPC1, it appears that word processing and memory encoding are facilitated more for positive and neutral words than for pain-related and negative words due to the former’s smaller LPC1 amplitudes in the left regions. In addition, also during LPC1, emotional word evaluation appears more facilitated for pain-related and negative words than for positive and neutral words due to the former’s smaller LPC1 amplitudes in the right regions.

For the subsequent LPC2, the pattern appears diametrical because of the lateralization change compared to LPC1. In LPC2, word processing and memory encoding are more facilitated for pain-related and negative words than positive and neutral words. In addition, emotional word evaluation appears more facilitated for positive and neutral words than pain-related and negative words. While the exact moment for these continued processes of detailed word evaluation and memory encoding differs between positive valent words and negative words, the processes per se remain the same. This observation is very interesting since no studies in the field have investigated different LPC time frames, and most did not consider lateralization processes. This omission may be the reason for the very heterogenous results of other studies.

### Early components

Unlike the late ERPs mentioned above, we found no effects of emotionally valenced words on early components. This finding is consistent with other studies that found no such effects for the N1 [12–14], the P2 [18, 19], and the P3 [8, 19, 22]. Some studies argued that early ERP components are unsuitable for distinguishing valenced and non-valenced words and are too confounded with arousal effects [8, 12, 13]. Some even attributed early ERPs after the presentation of valenced words to artifacts that arise when confounding dimensions (physical or linguistic) are not controlled [13]. Hinojosa et al. [21] found electrophysiological effects of valenced words for late components but of valenced pictures for early and late components. Therefore, they argued that words might not be the best stimulus to elicit early ERP effects. Overall and consistent with Citron [1], later ERPs such as the N400 and LPC more precisely distinguish between different emotional valences of words than early components such as the N1, P2, or P3. Especially for early components, results are very mixed and contradictory, as described in the introduction. It is argued that later components reflect higher cognitive processes involving stimulus evaluation and access to memory or mental representation and are, therefore, more suitable to distinguish between valenced words [1]. Early, more stimulus-driven components might not discriminate along the valence dimension as well as later components. This difference could explain the absence of valence effects in the early ERPs.

Despite the absence of valence effects in the early ERPs, we found prime-specific effects for N1, P2, and N400. We found larger N1 amplitudes in the left frontal region and N400 amplitudes in the right frontocentral region with than without painful primes. Frontal P2 amplitudes were larger without than with painful primes. It is important to remember that the interval between the onset of the painful primes and the analyzed ERPs varies because of the variable jitter between the onset of the painful prime and the onset of word presentation. Therefore, it is difficult to interpret the prime-specific results. Early components are often considered more stimulus-driven and related to attentional processes [1]. The prime-specific effects we found may be some kind of arousal effect. Early components are known to reflect arousal rather than valence effects [1, 8, 13, 15]. Ongoing painful primes (like in our experiment) might superpose early effects of word processing and trigger a higher arousal level because the painful primes might be more salient than the visually presented words. This process might allocate more attentional resources during painful priming and result in the early ERP effects we discovered in our experiment.

### Prime*Category

Contrary to our expectations, we found no Prime*Category or Prime*Category*Region interactions for the electrophysiological potentials. Therefore, we could not find pain-specific priming effects on valence-affected ERPs. At the behavioral level, this pain-specific priming effect on valence ratings occurred. Ratings were more negative with than without painful primes, with larger effects for pain-related, negative, and positive words and smaller effects for neutral words. These behavioral effects are consistent with previous findings for painful [26] and auditory [51] primes (for a detailed discussion of these effects, see [26]). Our results indicate that the Prime*Category interaction effect does not directly translate from the behavioral to the electrophysiological level, possibly due to several reasons. There might be two time-specific pathways, one for the effect of prime and one for the effect of category. The Prime*Region interaction might reflect early frontocentral activity patterns specific to the prime vs. no prime condition. The later occurring Category*Region interaction might reflect a later (also frontocentral) pathway that more specifically processes the word category.

**Fig 8:**
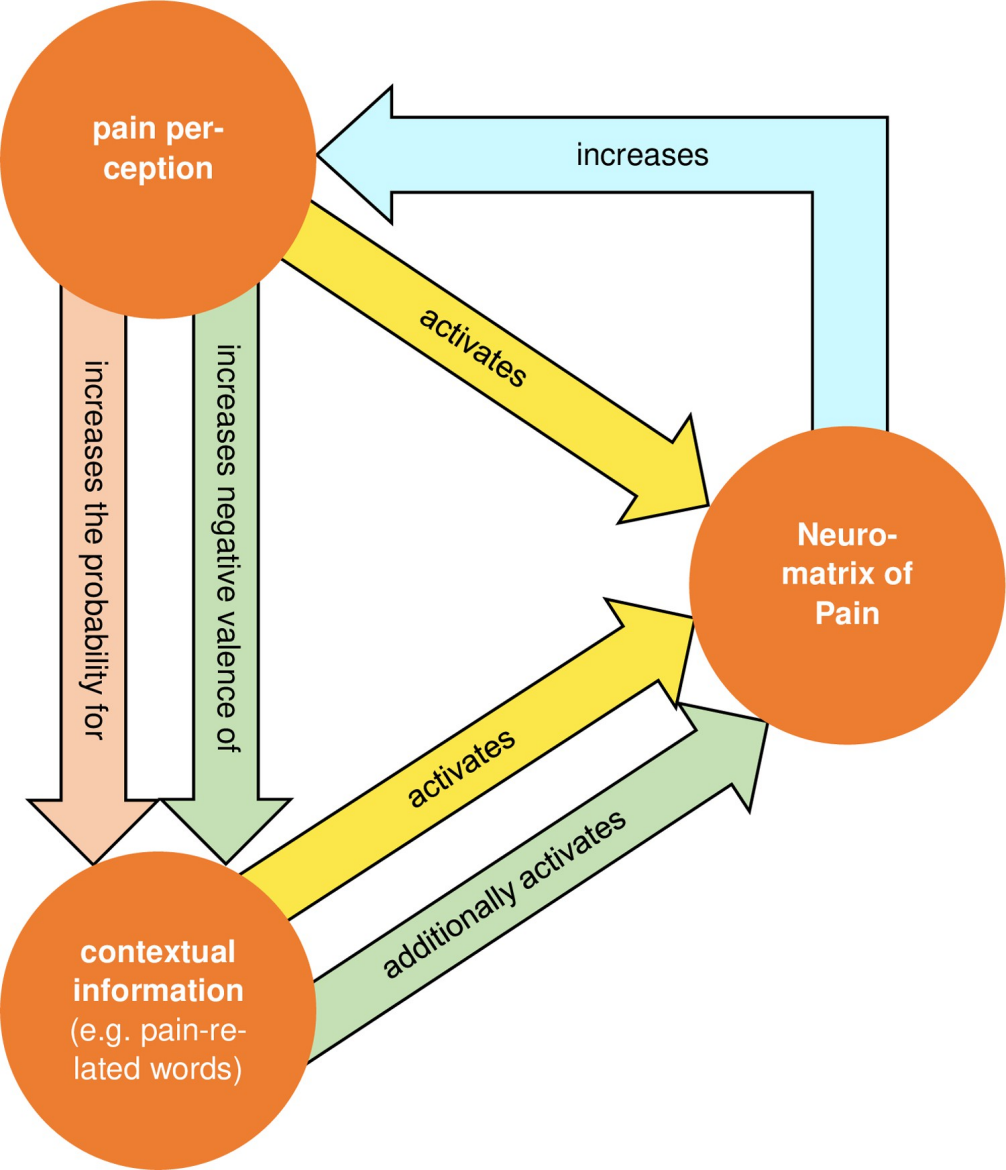
Extended hypothetical vicious circle model. The relationships in the green arrows have been added. Additional explanations in the text. Modified according to Richter and Weiß [54].

The Prime*Category interaction in the behavioral data might be a combination of these two pathways. The frontocentral activity pattern is consistent with another study that found a similar pattern for linguistic primes and painful targets [27]. This underlines the possibility of a vicious circle proposed in a previous paper [26]. It describes processes for the origin, development, and maintenance of chronic pain (Fig 8). The model shows that pain stimuli increase the probability for the occurrence of pain-specific contextual information, e.g. pain-related words during the contact with a physician. This pain-specific contextual information together with the painful stimuli lead to activation of the neuromatrix of pain [52, 53]. This increased activation of the neuromatrix of pain results in an increased perception of pain, which in turn again leads to an increased probability for the occurrence of pain-specific contextual information and an activation of the pain matrix. In addition, pain sensations do not just only increase the probability of the occurrence of pain-specific contextual information (e.g. pain-related words) but change the valence of the contextual information itself (e.g. the valence of pain-related words becomes more negative). This intensifies the following activation of the pain matrix and, thus, the perception of pain, resulting in an acceleration of the vicious circle which may lead to a downward spiral. In other words, felt pain might be described by patients with pain-related words, which increases internal pain ratings. In turn, these increased pain ratings might lead to more negative internal valence ratings of pain-related words, which again might lead to an increase of internal pain ratings. In this study the electrophysiological correlates of parts of this vicious circle were identified.

Another explanation for the discrepancy between electrophysiological and behavioral results might be the relatively small effect size of the Prime*Category interaction effect for the behavioral data. The size of the effect might be too small to translate into a corresponding electrophysiological effect. Kissler and Bromberek-Dyzman [10] found audiovisual-specific priming effects on some valence-affected electrophysiological potentials (such as N1 and LPC) but not others (such as N400). However, they reported partly prime-congruent and partly prime-incongruent effects. More studies are needed to investigate further the electrophysiological representations of pain-specific priming effects on the valence of words.

### Limitations and future research

The absence of effects of emotionally valenced words on early components may be due to superposing arousal effects of the painful primes. Future studies should consider this and use different intensity levels for the pain stimuli [31]. More intense pain stimuli might elicit higher arousal effects than less intense stimuli. With a variation in intensity level, it could be possible to better discriminate between arousal and valence effects in early components. We argued that heterogeneous results for later components, such as the LPC, found in other studies might be due to a low temporal resolution. That was the reason we used two consecutive LPC time frames. In addition, unlike other studies, we found more detailed lateralization effects, especially for later components. Therefore, future research should continue to use higher resolutions in EEG data analyses, both temporal (especially for the LPC) and spatial. Furthermore, it would be interesting to design a study comparing different word classes (adjectives, nouns, and verbs) and different tasks (lexical decision, silent reading, and written word identification) to better control for their respective effects. However, such a study would be very complex and challenging to perform.

During the experiment each word stimulus was repeated 10 times. This could have caused repetition effects (i.e., amplitude reduction) on the electrophysiological components, which could have diluted the effect of other factors. Future studies should use a larger number of unique stimuli per word category with fewer repetitions for each word without neglecting other linguistic features such as word length, number of syllables, arousal, valence, use in everyday language, and relatedness to one of the categories.

Some evidence shows that written words are more reliable in priming semantic access than spoken words, pictures, or environmental sounds [55]. However, other evidence shows that non-verbal stimuli elicit stronger electrophysiological potentials than words [2]. Future research should consider this when further investigating the correlation between priming effects and electrophysiological potentials. Another interesting question requiring further research is the differences in electrophysiological priming effects between healthy individuals and patients with chronic pain. Sitges et al. [19] reported ERP effects between pain-related and positive words for patients with chronic pain but not for healthy controls. Therefore, these valence-related word effects might also be differentially affected by painful primes.

## Conclusions

In conclusion, this study found larger ERP amplitudes for negative and pain-related words than for positive and neutral words in later components (N400, LPC1, and LPC2), mostly in the frontal regions. We found an activity shift between LPC1 and LPC2 in the frontal regions. LPC1 amplitudes were larger for pain-related and negative words than for neutral and positive words in the left frontocentral regions. However, the opposite effect was observed in the right frontocentral region. This frontocentral activity pattern in LPC1 then shifts to the contralateral regions in LPC2. Early components such as N1, P2, and P3 appear unaffected by word category. We found prime-specific effects for early ERPs (N1, P2, and N400), including larger N1 amplitudes in the left frontal region and N400 amplitudes in the right frontocentral region with than without painful primes but larger frontal P2 amplitudes without than with painful primes. This finding partly confirms our hypothesis that later components, such as the N400 and LPC, would show larger ERP amplitudes for emotionally valenced words.

## Supporting information

S1 Table Corrected p-values for contrasts regarding main effect of the *Region* factor. (PDF)

S1 File (PDF)

S2 File (DOCX)
