# Supplementary material for: Neural correlates of word processing influenced by painful primes
Source: PLoS One. 2024 Jan 19;19(1):e0295148. doi: 10.1371/journal.pone.0295148 (PMC10798507; doi:10.1371/journal.pone.0295148)
Supplement: S1 Table — (PDF) [file pone.0295148.s001.pdf]

S1 Table. Corrected p-values for contrasts regarding main effect of the Region factor.

N1:

Data are reported as mean (SE); ***: p < 0.001, **: p < 0.01, *: p < 0.05.

	Left Frontal	Middle Frontal	Right Frontal	Left Central	Middle Central	Right Central	Left Parietal	Middle Parietal	Right Parietal	Occipital
Frontopolar (-0.79 ± 0.15 μV)	.009**	.659	<.001***	.174	<.001***	<.001***	.028*	<.001***	<.001***	<.001***
Left Frontal (-1.35 ± 0.12 μV)		.014*	<.001***	<.001***	<.001***	<.001***	<.001***	<.001***	<.001***	<.001***
Middle Frontal (-0.93 ± 0.13 μV)			<.001***	.311	<.001***	<.001***	.008**	<.001***	<.001***	<.001***
Right Frontal (-0.01 ± 0.14 μV)				.152	.533	<.001***	.312	.247	.015*	.056
Left Central (-0.52 ± 0.09 μV)					.247	<.001***	<.001***	<.001***	<.001***	<.001***
Middle Central (-0.11 ± 0.11 μV)						<.001***	.128	<.001***	<.001***	.004**
Right Central (0.65 ± 0.08 μV)							.234	.830	.343	.117
Left Parietal (0.32 ± 0.21 μV)								.176	.059	.005**
Middle Parietal (0.61 ± 0.14 μV)									.190	.212
Right Parietal (1.07 ± 0.19 μV)										.907
Occipital (1.05 ± 0.23 μV)										

P2:

Data are reported as mean (SE); ***: p < 0.001, **: p < 0.01, *: p < 0.05.

	Left Frontal	Middle Frontal	Right Frontal	Left Central	Middle Central	Right Central	Left Parietal	Middle Parietal	Right Parietal	Occipital
Frontopolar (2.41 ± 0.20 μV)	<.001***	.787	<.001***	<.001***	<.001***	<.001***	<.001***	<.001***	<.001***	<.001***
Left Frontal (1.71 ± 0.17 μV)		<.001***	.022*	<.001***	.004**	<.001***	<.001***	<.001***	<.001***	<.001***
Middle Frontal (2.43 ± 0.19 μV)			<.001***	<.001***	<.001***	<.001***	<.001***	<.001***	<.001***	<.001***
Right Frontal (1.04 ± 0.17 μV)				<.001***	.821	<.001***	<.001***	<.001***	<.001***	<.001***
Left Central (-0.49 ± 0.11 μV)					<.001***	.197	<.001***	.014*	<.001***	<.001***
Middle Central (1.00 ± 0.13 μV)						<.001***	<.001***	<.001***	<.001***	<.001***
Right Central (-0.72 ± 0.10 μV)							<.001***	.051	<.001***	<.001***
Left Parietal (-2.39 ± 0.24 μV)								<.001***	.300	.280
Middle Parietal (-1.19 ± 0.16 μV)									<.001***	.003**
Right Parietal (-2.16 ± 0.24 μV)										.884
Occipital (-2.13 ± 0.28 μV)										

P3:

Data are reported as mean (SE); ***: p < 0.001, **: p < 0.01, *: p < 0.05.

	Left Frontal	Middle Frontal	Right Frontal	Left Central	Middle Central	Right Central	Left Parietal	Middle Parietal	Right Parietal	Occipital
Frontopolar (-1.58 ± 0.15 µV)	.009**	<.001***	.752	<.001***	<.001***	<.001***	<.001***	<.001***	<.001***	<.001***
Left Frontal (-0.98 ± 0.16 µV)		.800	.800	<.001***	<.001***	<.001***	<.001***	<.001***	<.001***	<.001***
Middle Frontal (-1.17 ± 0.14 µV)			.800	<.001***	<.001***	<.001***	<.001***	<.001***	<.001***	<.001***
Right Frontal (-1.33 ± 0.15 µV)				<.001***	<.001***	<.001***	<.001***	<.001***	<.001***	<.001***
Left Central (-0.23 ± 0.12 µV)					.800	.800	<.001***	<.001***	<.001***	<.001***
Middle Central (0.04 ± 0.10 µV)						.800	<.001***	<.001***	<.001***	<.001***
Right Central (0.09 ± 0.10 µV)							<.001***	<.001***	<.001***	<.001***
Left Parietal (1.50 ± 0.17 µV)								.800	.036*	<.001***
Middle Parietal (1.82 ± 0.16 µV)									.800	.002**
Right Parietal (2.13 ± 0.20 µV)										.275
Occipital (2.61 ± 0.23 µV)										

N400:

Data are reported as mean (SE); ***: p < 0.001, **: p < 0.01, *: p < 0.05.

	Left Frontal	Middle Frontal	Right Frontal	Left Central	Middle Central	Right Central	Left Parietal	Middle Parietal	Right Parietal	Occipital
Frontopolar (-2.65 ± 0.15 µV)	<.001***	<.001***	<.001***	<.001***	<.001***	<.001***	<.001***	<.001***	<.001***	<.001***
Left Frontal (-0.74 ± 0.14 µV)		<.001***	.003**	<.001***	.920	<.001***	<.001***	<.001***	<.001***	<.001***
Middle Frontal (-2.18 ± 0.13 µV)			<.001***	<.001***	<.001***	<.001***	<.001***	<.001***	<.001***	<.001***
Right Frontal (-1.43 ± 0.11 µV)				<.001***	.006**	<.001***	<.001***	<.001***	<.001***	<.001***
Left Central (0.77 ± 0.09 µV)					<.001***	.007**	<.001***	.867	<.001***	<.001***
Middle Central (-0.95 ± 0.11 µV)						<.001***	<.001***	<.001***	<.001***	<.001***
Right Central (0.33 ± 0.08 µV)							<.001***	.006**	<.001***	<.001***
Left Parietal (1.89 ± 0.14 µV)								<.001***	.703	.005**
Middle Parietal (0.79 ± 0.11 µV)									<.001***	<.001***
Right Parietal (1.84 ± 0.13 µV)										<.001***
Occipital (2.30 ± 0.15 µV)										

LPC1:

Data are reported as mean (SE); ***: p < 0.001, **: p < 0.01, *: p < 0.05.

	Left Frontal	Middle Frontal	Right Frontal	Left Central	Middle Central	Right Central	Left Parietal	Middle Parietal	Right Parietal	Occipital
Frontopolar (0.71 ± 0.19 μV)	.040*	.375	.008**	.353	.451	<.001***	<.001***	<.001***	<.001***	<.001***
Left Frontal (1.36 ± 0.15 μV)		.007**	<.001***	<.001***	<.001***	<.001***	<.001***	<.001***	<.001***	<.001***
Middle Frontal (0.82 ± 0.11 μV)			<.001***	.400	<.001***	<.001***	<.001***	<.001***	<.001***	<.001***
Right Frontal (0.14 ± 0.15 μV)				.177	.530	<.001***	.007**	.117	<.001***	<.001***
Left Central (0.46 ± 0.11 μV)					.140	<.001***	<.001***	<.001***	<.001***	<.001***
Middle Central (0.26 ± 0.09 μV)						<.001***	<.001***	<.001***	<.001***	<.001***
Right Central (-0.59 ± 0.10 μV)							.228	.528	<.001***	.005**
Left Parietal (-0.79 ± 0.12 μV)								.117	<.001***	<.001***
Middle Parietal (-0.49 ± 0.12 μV)									<.001***	<.001***
Right Parietal (-1.38 ± 0.14 μV)										.227
Occipital (-1.25 ± 0.15 μV)										

LPC2:

Data are reported as mean (SE); ***: p < 0.001, **: p < 0.01, *: p < 0.05.

	Left Frontal	Middle Frontal	Right Frontal	Left Central	Middle Central	Right Central	Left Parietal	Middle Parietal	Right Parietal	Occipital
Frontopolar (2.81 ± 0.27 μV)	<.001***	<.001***	<.001***	<.001***	<.001***	<.001***	<.001***	<.001***	<.001***	<.001***
Left Frontal (0.30 ± 0.23 μV)		<.001***	<.001***	<.001***	.692	.641	<.001***	.021*	<.001***	<.001***
Middle Frontal (1.73 ± 0.15 μV)			.947	<.001***	<.001***	<.001***	<.001***	<.001***	<.001***	<.001***
Right Frontal (1.74 ± 0.17 μV)				<.001***	<.001***	<.001***	.007**	<.001***	<.001***	<.001***
Left Central (-0.92 ± 0.11 μV)					<.001***	<.001***	<.001***	.692	.048*	<.001***
Middle Central (0.60 ± 0.16 μV)						<.001***	<.001***	<.001***	<.001***	<.001***
Right Central (-0.14 ± 0.10 μV)							<.001***	<.001***	<.001***	<.001***
Left Parietal (-1.59 ± 0.13 μV)								<.001***	.641	<.001***
Middle Parietal (-0.71 ± 0.12 μV)									<.001***	<.001***
Right Parietal (-1.45 ± 0.16 μV)										<.001***
Occipital (-2.12 ± 0.18 μV)										
