# Supplementary material for: Neural correlates of word processing influenced by painful primes
Source: PLoS One. 2024 Jan 19;19(1):e0295148. doi: 10.1371/journal.pone.0295148 (PMC10798507; doi:10.1371/journal.pone.0295148)
Supplement: S1 File — (PDF) [file pone.0295148.s002.pdf]

seit 1548

Friedrich-Schiller-Universität Jena

Ethikkommission der Fakultät für Sozial- und Verhaltenswissenschaften
Ethical Commission of the Faculty of Social and Behavioural Sciences

Univ.-Prof. Dr. mult. Nikolaus Knoepffler (Präsident, chairman)

Bestätigung

Hiermit bestätige ich als Präsident der Ethikkommission der Fakultät für Sozial- und Verhaltenswissenschaften der Friedrich-Schiller-Universität Jena, dass der Ethikantrag zur Studie „*Konditionierung und Extinktion von Schmerzantworten auf neutrale Adjektive*", der von Herrn Prof. Dr. Thomas Weiß (Institut für Psychologie, Universität Jena) präsentiert wurde, als ethisch unbedenklich qualifiziert wird.

Der Vorgang ist unter der Nummer FSV 15/17 abgelegt.

Wir wünschen allen Beteiligten ein erfolgreiches Forschungsvorhaben.

Jena, den 16.12.2015

N. Knoepffler

Prof. Dr. mult. Nikolaus Knoepffler

Adresse: Zwätzengasse 3, 07743 Jena, T. 03641/945800, E-Mail: n.knoepffler@uni-jena.de
