# Supplementary material for: Neural correlates of word processing influenced by painful primes
Source: PLoS One. 2024 Jan 19;19(1):e0295148. doi: 10.1371/journal.pone.0295148 (PMC10798507; doi:10.1371/journal.pone.0295148)
Supplement: S2 File — (DOCX) [file pone.0295148.s003.docx]

**Requested English Translation of the Vote to our research on Priming of word processing by painful primes**

Friedrich Schiller University Jena

Ethical Commission of the Faculty of Social and Behavioural Sciences

Univ.-Prof. Dr. mult. Nikolaus Knoepffler (Prasident, chairman)

**Confirmation**

As President of the Ethical Commission of the Faculty of Social and Behavioral Sciences at the Friedrich Schiller University Jena, I hereby confirm that the ethics application for the study

***"Priming of word processing through pain stimuli"***

submitted by Prof. Dr. Thomas Weiss (Institute for Psychology, University of Jena) was presented and qualified as being ethically harmless.

The process is registered as FSV 14/04.

We wish all participants a successful research project.

(Sign)

Prof. Dr. mult. Nikolaus Knoepffler
